# Epetraborole, a leucyl-tRNA synthetase inhibitor, demonstrates murine efficacy, enhancing the *in vivo* activity of ceftazidime against *Burkholderia pseudomallei*, the causative agent of melioidosis

**DOI:** 10.1371/journal.pntd.0011795

**Published:** 2023-11-27

**Authors:** Jason E. Cummings, Christopher S. Lunde, M. R. K. Alley, Richard A. Slayden

**Affiliations:** 1 Microbiology, Immunology and Pathology, Colorado State University, Fort Collins, Colorado, United States of America; 2 AN2 Therapeutics, Menlo Park, California, United States of America; Tufts Medical Center, UNITED STATES

## Abstract

*Burkholderia pseudomallei* is the causative agent of melioidosis, which is increasingly being reported worldwide. Mortality rates as high as 40% have been reported based on clinical patient outcomes in the endemic areas of Australia and Thailand. Novel therapies are needed to reduce treatment duration and adverse effects and improve treatment outcomes. Epetraborole, a novel antibiotic, targets leucyl-tRNA synthetase (LeuRS), an essential enzyme that catalyzes the attachment of leucine to transfer RNA. Epetraborole was evaluated for *in vitro* activity and efficacy in a murine model to assess clinical relevance against *Burkholderia pseudomallei* infections for possible treatment of melioidosis. Epetraborole was tested against 13 clinically derived and three reference *B*. *pseudomallei* strains that have a broad spectrum of susceptibilities to the standard-of-care (SoC) drugs for melioidosis, which showed that epetraborole exhibited minimal inhibitory concentrations of 0.25–4 μg/mL. *Ex vivo* studies using THP-1 macrophages confirmed the potency of epetraborole and demonstrated synergy between epetraborole and ceftazidime. In the acute pulmonary murine infection model of melioidosis, epetraborole demonstrated equivalent efficacy when delivered orally or subcutaneously, which compared well with the standard-of-care drug ceftazidime. In addition, adding epetraborole to ceftazidime significantly improved antimicrobial activity in this animal model. This work warrants further exploration of epetraborole as a candidate for treating melioidosis and substantiates LeuRS as a clinically relevant drug target in *B*. *pseudomallei*.

## Author summary

Our study suggests the repurposing of epetraborole for the treatment of the often rapidly progressing and fatal disease melioidosis is warranted. Melioidosis is caused by the bacterial pathogen *Burkholderia pseudomallei*, commonly found in Southeast Asia, Australia, Central and South America, Mexico, and recently in the Southern United States. The current clinical treatment of melioidosis involving multiple drugs is extensive, and a portion of infections will persist or relapse after the completion of treatment. Resistance to the majority of antibiotics limits the ability to treat and manage the disease effectively. However, *Burkholderia pseudomallei* is an under-recognized emerging pathogen with a general lack of resources and interest to drive research for new treatments. Epetraborole was initially identified with *in vitro* and *in vivo* activity against various medically important Gram-negative bacterial infections and was advanced to treat complicated urinary tract infections. Accordingly, we have assessed the use of epetraborole to treat melioidosis in a mouse model when administered alone or in combination with the current clinically used drug ceftazidime.

## Introduction

*Burkholderia pseudomallei*, the causative agent of the tropical disease melioidosis, is an infectious bacterium that causes acute fatal disease in humans, which was first described in 1912 in Rangoon by Whitmore and Khrisnaswami [1]. Although most human cases worldwide are identified in Southeast Asia and Australia, with Thailand reporting the highest mortality rate of ~39%, followed by Australia at ~14%, there is mounting evidence that *B*. *pseudomallei* is an under-recognized emerging pathogen in Central and South America, Mexico, and more recently the Southern United States [2–7]. Until recently, most cases diagnosed in the United States could be attributed to travel to areas where *B*. *pseudomallei* is endemic [3]. Indeed, recent research on the incidence of *B*. *pseudomallei* suggests the disease is endemic in many countries throughout the Americas [7]. The wide geographic distribution, high morbidity, and mortality necessitate continuing drug discovery efforts [2,8].

Treatment of melioidosis is lengthy compared to other Gram-negative bacterial infections, with a minimum of 2 weeks of IV therapy of ceftazidime or meropenem followed by 3–6 months of oral therapy with trimethoprim-sulfamethoxazole [9]. Although this treatment regimen is used clinically, mortality remains high, and a percentage of successfully treated infections will persist or relapse after the completion of treatment [10,11]. The mortality, persistence, and relapse observed after treatment have been attributed to the poor intracellular activity of the currently used standard-of-care (SoC) drugs toward facultative intracellular growth of the organism as being a critical aspect of the pathobiology of the disease [12–16]. Another significant impact on drug susceptibility is the host response during infection, which has been observed to decrease the potency of ceftazidime [17]. Transient *in vivo* resistance mechanisms and natural resistance due to efflux pumps render most drugs ineffective against *Burkholderia* infections and limit the ability to treat and manage the disease [8,10,18–21].

The clinical drug candidate, epetraborole, is a boron-containing inhibitor of the bacterial leucyl-tRNA synthetase (LeuRS). Epetraborole was initially identified with *in vitro* and *in vivo* activity against Gram-negative bacteria. Epetraborole was advanced to a phase 2 clinical trial for complicated urinary tract infections. These studies demonstrated that epetraborole has broad-spectrum potential and LeuRS is a clinically relevant drug target. LeuRS is an essential enzyme for protein synthesis that attaches leucine to tRNA^Leu^. In addition to this aminoacylation active site, LeuRS has an editing site that hydrolyzes incorrect amino acids from its cognate tRNA [22]. Epetraborole inhibits LeuRS by trapping tRNA^Leu^ in the editing active site via its boron atom binding to the cis-diols of the terminal adenosine of tRNA^Leu^ [23], which prevents tRNA^Leu^ from being leucylated by the aminoacylation site. Thus, the inhibition of Leu-tRNA^Leu^ synthesis ultimately leads to a block in protein synthesis. In addition to epetraborole’s novel mechanism of action, it has physiochemical properties appropriate for a melioidosis drug candidate. Epetraborole has a small molecule weight (MW 237), high polarity with a LogD_7.4_ of -0.23, very low plasma protein binding, and excellent cell penetration, including human alveolar macrophages [24]; these attributes justify the exploration of epetraborole’s activity against *B*. *pseudomallei*.

Accordingly, we assessed the *in vitro* activity and *in vivo* efficacy of epetraborole against *B*. *pseudomallei* as monotherapy and in combination with the standard of care (SoC) drug ceftazidime. Epetraborole demonstrated potency against a panel of laboratory reference and clinically derived *B*. *pseudomallei* strains and efficacy in *in vitro*, *ex vivo*, and an acute animal model of melioidosis. The assessment of epetraborole against clinically derived strains and in efficacy models allowed us to directly assess the potential use of epetraborole to treat clinically relevant infections administered alone or in combination with ceftazidime. These data demonstrate that further exploration of epetraborole in the therapy of melioidosis is warranted, which further supports LeuRS as a clinically relevant drug target.

## Materials and methods

### Ethics statement

All studies performed at Colorado State University were conducted in a BSL3 facility dedicated to bacterial pathogen work under the approvals and management of the Biosafety Official. Studies were approved by the Institutional Biosafety Committee and the Institutional Animal Care and Use Committee and performed under approvals PARF 17-095B and IACUC protocol 3796.

### Bacteria and minimum inhibitory concentration determination

*B*. *pseudomallei* strains were grown to an OD_600_ of ~0.6 and were frozen in 10% (v/v) glycerol at -80°C as standard bacterial stocks for these studies. For each evaluation, bacteria were prepared fresh by growth from the standard Luria-Bertani (LB) Agar stocks at 37°C for 48–72 hrs. Bacteria recovered from the LB plates were used to inoculate 10 mL LB Broth. Broth cultures were then incubated for 18 hrs at 37°C, passed 1:100, and incubated for an additional 6 hrs at 37°C. Bacteria were then diluted into 1x10^6^ colony forming units (CFU)/mL concentration in cation-adjusted Mueller-Hinton (caMH) Broth (BD, Franklin Lakes, NJ), and 50 μL was added to each well for each drug plate for a final inoculum of 5x10^5^ CFU/mL. For MIC determination, the concentration range tested for epetraborole and ceftazidime was 0.03–64 μg/mL in caMH broth. MIC plates were incubated at 37°C for 18 hrs, at which time MIC was determined per CLSI guidelines [25]. The MIC value for epetraborole and ceftazidime with the QC strain *P*. *aeruginosa* ATCC 27853 was 2 and 4 μg/mL, respectively, which is within their QC ranges.

### Burkholderia *ex vivo* model of efficacy

Infected THP-1 macrophages (American Type Culture Collection, Manassas, VA) were used to assess the intracellular effectiveness of these compounds. 2.5x10^5^ activated THP-1 cells per well in a 24-well tissue culture plate were cultured in a complete medium that consisted of Roswell Park Memorial Institute (RPMI) (Invitrogen) and 10% (v/v) fetal bovine serum (HyClone, Logan, UT). THP-1 cells were incubated at 37°C with 5% CO_2_ for 24 hours (hrs). Bacteria were added to THP-1 cells at a multiplicity of infection of 10 CFU per cell in a 0.5 mL media. The plates were centrifuged at 2,400 X g for 2 min and placed at 37°C/5% CO_2_ to incubate for 2 hrs. The supernatant was removed, and each well was washed twice with 2 mL phosphate-buffered saline (PBS, Sigma-Aldrich, St. Louis, MO). Epetraborole HCl (AN2 Therapeutics) and ceftazidime (Sigma-Aldrich) were added to each well at 8, 4, 2, 1, 0.5, and 0.25 μg/mL in complete media in triplicate. Media only were added to three wells to serve as a negative drug control. Plates were incubated for 24 hrs, and cells were observed for signs of infection. The cells were washed thrice with 2 mL PBS and lysed with 1 mL sterile 0.05% SDS/water. Each well was thoroughly mixed/scraped and incubated at room temperature for 7 minutes to ensure complete cell lysis. Lysates were then serially diluted at 1:10, and inoculum plated from the neat, 10^−1^, 10^−2^, 10^−3,^ and 10^−4^ dilutions onto LB agar plates. Plates were incubated for 48 hrs at 37°C. Colonies from each plate were counted, and Log_10_ CFU/mL lysate was calculated.

### Pharmacokinetic analysis

Epetraborole pharmacokinetics were assessed in female BALB/c mice by Quintara Discovery Inc. (Hayward, CA 94545). Epetraborole formulation and bioanalysis were performed as described by Hernandez et al. [23]. Eighteen Balb/c female mice, 7–8 weeks old, were dosed once at 30 mg/kg either orally (PO) or subcutaneously (SC), and plasma concentrations of epetraborole were measured.

### Burkholderia animal model of infection

Acute *B*. *pseudomallei* mouse model of disease for efficacy evaluation used 7–9 week-old BALB/c female mice (Charles River Laboratories, Wilmington, MA), which were challenged by intranasal infection with 5,000 CFU/mouse *B*. *pseudomallei* 1026b. Animals were anesthetized with a mixture of 100 mg/kg ketamine and 10 mg/kg xylazine delivered intraperitoneally before receiving the inoculum in a 20 μL volume in alternating nostrils. Ceftazidime was formulated in PBS (pH 7.4) to be delivered subcutaneously (SC) at 200, 125, and 25 mg/kg twice a day (BID). Epetraborole was formulated for injection in water (pH 5) to be given SC at 300, 100, and 30 mg/kg or orally (PO) at 30, 15, and 5 mg/kg BID. Dosing for mice assessed with combinatorial drug therapy was 200/30, 125/15, and 25/5 mg/kg ceftazidime and epetraborole. Drugs were delivered 2 hrs post-infection, and repeated doses were given every 24 hrs. The number of viable bacteria in the lung and spleen was determined at 60 hrs post-exposure by plating serial 10-fold dilutions of homogenates onto LB agar and incubating for 48 hrs at 37°C. Bacterial burden was assessed using a one-way analysis of variance (ANOVA) followed by Tukey’s multiple comparison tests with significance determined by a P-value < 0.05.

### Delayed dosing efficacy evaluation in acute *B*. *pseudomallei* mouse model of disease

7–9 week-old BALB/c female mice were challenged by intranasal infection with 5,000 CFU/mouse *B*. *pseudomallei*. Animals were anesthetized with a mixture of 100 mg/kg ketamine and 10 mg/kg xylazine delivered intraperitoneally before receiving the inoculum in a 20 μL volume in alternating nostrils. Ceftazidime was formulated in PBS (pH 7.4) to be delivered subcutaneously (SC) at 250 mg/kg for every 4hrs, and epetraborole was formulated for injection in water (pH 5) to be delivered intraperitoneally (IP) at 200 and 100 mg/kg and dosed 12 hours after infection. Combination therapy was assessed by dosing mice with 200 and 100 mg/kg epetraborole IP BID plus the ceftazidime regimen described above. All study drugs were administered 12 hours post-infection for delayed dosing efficacy evaluation. The number of viable bacteria in the lung and spleen was determined at 60 hrs post-exposure by plating serial 10-fold dilutions of homogenates onto LB agar and incubating for 48 hrs at 37°C. Bacterial burden was assessed using a one-way analysis of variance (ANOVA) followed by Tukey’s multiple comparison tests with significance determined by a P-value < 0.05.

## Results

### Epetraborole is a potent inhibitor of clinically derived *Burkholderia pseudomallei* strains

Epetraborole was screened for anti-burkholderia activity using a standard broth microdilution method [10]. Epetraborole and the comparator standard of care drug ceftazidime were first tested against the *B*. *pseudomallei* laboratory reference strains 1026b and K96243, the clinical representative susceptible strain DD503, and the drug efflux deficient *B*. *pseudomallei* strain Bp400 to determine the baseline inhibitory activity of epetraborole and assess as an efflux pump substrate. These tests revealed that epetraborole has a MIC range of 0.25–1 μg/mL across these strains, with DD503 and Bp400 being the most susceptible and the laboratory strains 1026b and K96243 being the least sensitive (**Table 1**). The *in vitro* activity of epetraborole was superior to the SoC drug ceftazidime, which has a MIC range of 2–4 μg/mL against these strains. The Bp400 strain, with the BepAB-OprB and AmrAB-OprA efflux pumps deleted, was only 4-fold more susceptible than its parental wild-type strain 1026b [26]. A secondary screen was performed against a diverse panel of clinically derived *B*. *pseudomallei* strains representative of the drug susceptibility spectrum associated with clinical infections [8]. Against these strains, epetraborole has a MIC range of 0.5 μg/mL to 4 μg/mL with a mode of 1 μg/mL. Ceftazidime has a MIC range of 2 μg/mL to 8 μg/mL with a mode of 4 μg/mL (**Table 1**). These studies confirm that epetraborole has potency across clinically representative strains comparable to the standard-of-care drugs currently used in the clinical treatment [8].

**Table 1:** The inhibitory concentration of Epetraborole against *Burkholderia pseudomallei*.

Strain	Epetraborole (μg/mL)	Ceftazidime (μg/mL)
**A. Laboratory Reference Strains**		
1026b	1	4
K96243	1	4
BP400	0.25	2
DD503	0.25	4
QC Strain *P*. *aeruginosa* ATCC 27853	2	4
**B. Diverse Panel of clinical isolates**		
China 3 (MP-H, NBL 104)	0.5	2
1106b	1	4
1710a	0.5	8
1710b	0.5	4
406e	1	4
MSHR435	2	8
MSHR668	1	2
MSHR465a	1	4
NCTC 6700	1	8
NCTC 7383	4	8
NCTC 7431	1	8
NCTC 10274	1	4
NCTC 10276	1	4
MIC Range	0.5–4	2–8
Modal MIC	1	4
MIC50	1	4
MIC90	2	8

### Epetraborole demonstrates efficacy in *ex vivo* and *in vivo Burkholderia* models of efficacy

**Fig 1:**
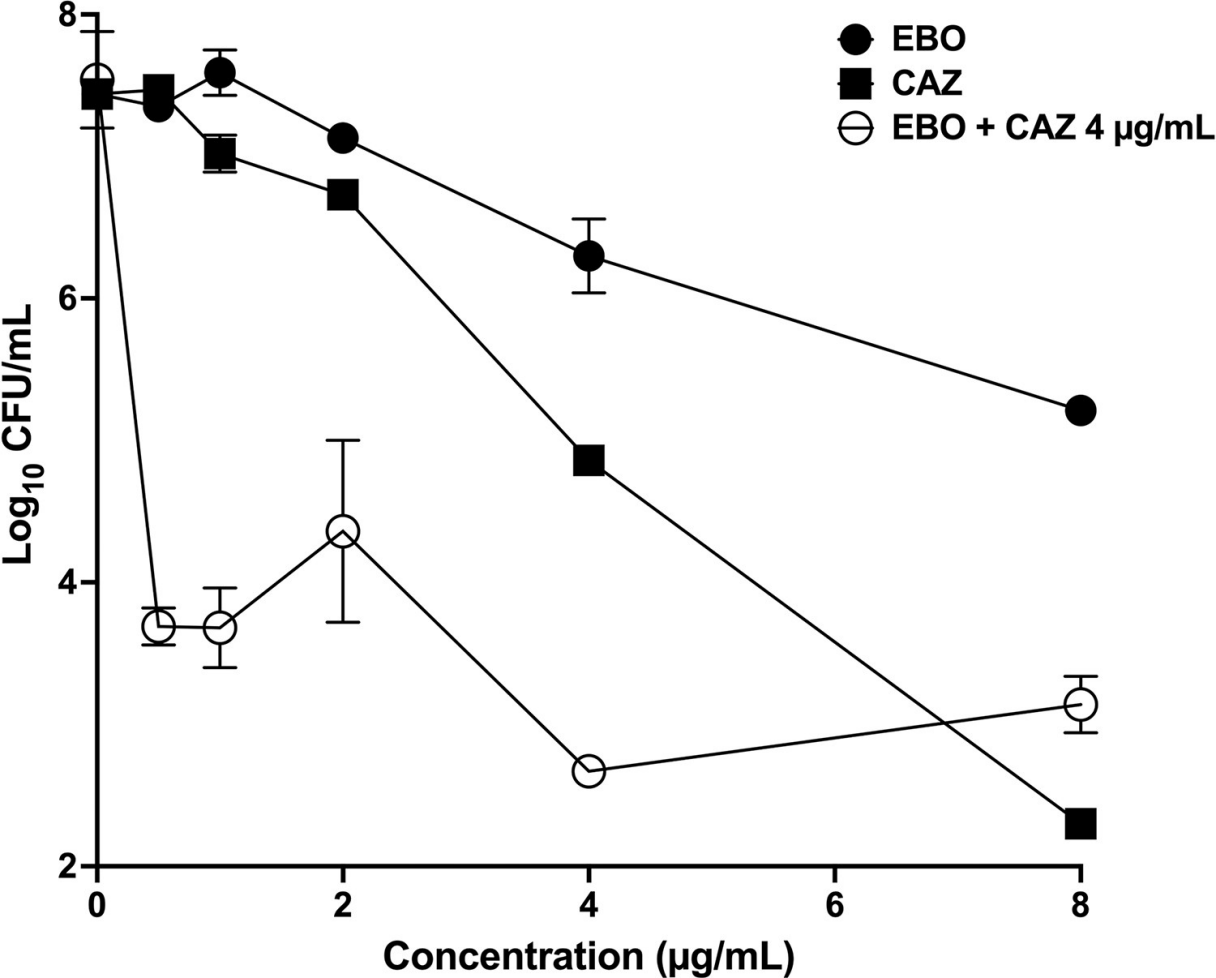
Epetroborale activity in *ex vivo* model of infection. Epetraborole (EBO) was evaluated against *B*. *pseudomallei* 1026b alone and in combination with ceftazidime at 4 mg/mL (EBO + CAZ). Ceftazidime (CAZ) was included as a comparative control. Growth inhibition was determined over a 0.25 to 8 μg/mL.epetraborole (EBO) concentration series For the combination treatment group, data points for 8, 4, 2, 1, 0.5 μg/mL are all significantly different from the untreated (0 μg/mL), and those data points are not significantly different from one another. All comparison analyses was performed with ANOVA.

Epetraborole was evaluated against *B*. *pseudomallei* 1026b in a *Burkholderia ex vivo* model. Growth inhibition was determined over a 0.25 to 8 μg/mL concentration series of epetraborole. Epetraborole inhibited intracellular growth of *B*. *pseudomallei* in a dose-dependent fashion, demonstrating a ~ 1.5 log_10_ reduction in CFUs at 4 μg/mL and ~ 2.5 log_10_ reduction in CFUs at 8 μg/mL (**Fig 1**). Similar dose-dependent decreases were observed for the control drug, ceftazidime, which demonstrated ~ 2 log_10_ reduction in CFUs at 4 μg/mL (**Fig 1**). Epetraborole was also evaluated as a co-therapeutic with ceftazidime to determine epetraborole’s use as a new agent in a combination therapy regimen. A dose of 4 μg/mL of ceftazidime was added to an epetraborole concentration series of 0.25–8 μg/mL, which showed that co-treatment of 1 μg/mL epetraborole with 4 μg/mL of ceftazidime showed a ~ 3.5 log_10_ reduction in CFUs. This reduction was more significant than the reduction observed for 4 μg/mL of ceftazidime alone (**Fig 1**). This demonstrates that the 1:4 combination of epetraborole and ceftazidime has an additive effect and can significantly reduce CFUs at lower concentrations than can be achieved with monotherapy. These studies also substantiate that epetraborole can gain access to the bacteria in macrophages.

**Fig 2:**
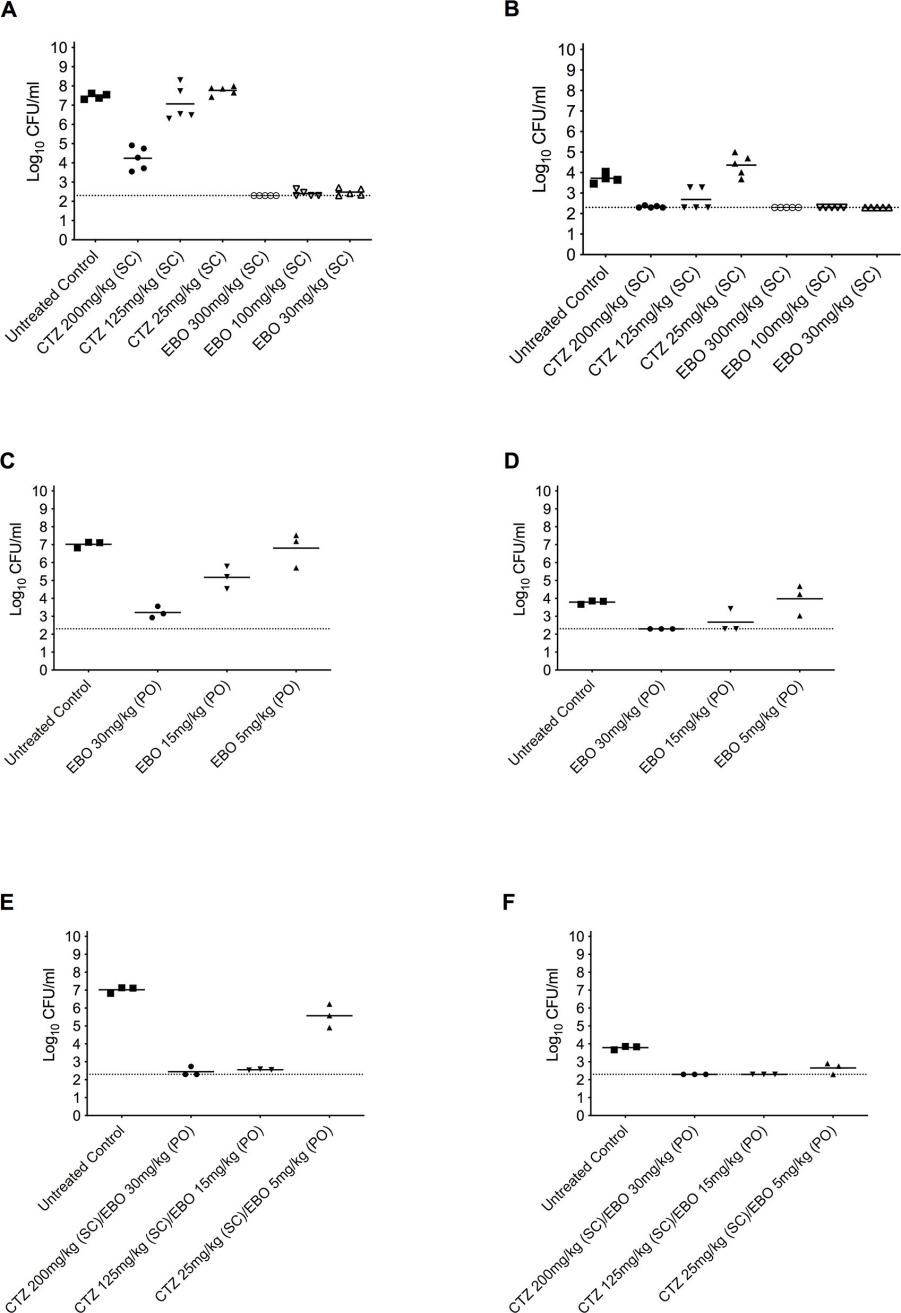
Efficacy of epetraborole in the acute *B*. *pseudomallei* animal infection model. Bacterial burdens in the lung (A, C, E) and spleen (B, D, F) during treatment with epetraborole delivered subcutaneously (A, B), orally (C, D), or in combination (E, F) compared to untreated control mouse organ burdens at 60h endpoint. Lower level of detection is indicated by dotted lines.

Epetraborole was tested in the acute *B*. *pseudomallei* animal infection model to determine efficacy. Initially, we chose to dose mice subcutaneously with 30, 100, or 300 mg/kg epetraborole BID, which equates to AUC_0-24_ of approximately 18.6, 62, 186 mg.h/L, assuming linearity from a single 30 mg/kg SC dose in a satellite PK group that yielded an AUC_0-24_ of 9.3 mg.h/L (**Table 2**). In comparison, in humans in 2 studies where epetraborole was dosed at 1500 mg q12h, it achieved exposures with an AUC_0-24_ of 146 mg.h/L [24]. The dose of 300 mg/kg BID of epetraborole exhibited a ~ 5 Log_10_ or greater CFU/mL reduction (P<0.05) in the lung compared to the untreated control group (**Fig 2A**). This CFU reduction was more significant than observed in mice treated with comparative doses of 25, 125, or 200 mg/kg ceftazidime BID, which resulted in a decrease of 0.5 to 4 Log_10_ CFU/mL in the lungs (P<0.05 for 200 mg/kg group). Dissemination of *B*. *pseudomallei* to the spleen was not detected in the epetraborole-treated groups, contrasting the observed dissemination in the lower ceftazidime-treated groups (**Fig 2B**). To determine epetraborole efficacy *via* the oral route, epetraborole was also delivered orally at 30 and 15 mg/kg doses of BID, which equates to an AUC_0-24_ of approximately 4.9 and 9.7 mg h/L assuming linearity from a single 30 mg/kg PO dose in a satellite PK group that yielded an AUC_0-24_ of 4.9 mg. h/L. These low oral doses of epetraborole reduced bacterial burden in the lungs by 1.8–3.8 Log_10_ CFU/mL (p<0.05) with the 30 mg/kg dose of epetraborole resulting in a ~ 3.5 Log_10_ CFU/mL reduction (P<0.05) in the lung compared to the untreated control group (**Fig 2C**). Oral treatment with 15 mg/kg of epetraborole resulted in a ~ 1.5 Log_10_ CFU/mL reduction (P<0.05) in the lung. Dissemination to the spleen was not observed in the animals treated orally with 30 mg/kg of epetraborole (**Fig 2D**).

**Table 2:** Epetraborole murine pharmacokinetic parameters.

	PO (30 mg/kg)	SC (30 mg/kg)
**Cmax (mg/L)**	2.11	4.66
**Tmax (h)**	0.5	0.5
**AUC**_**last**_ (h*mg/L)	4.87	9.26
**T**_**1/2**_ **(h)**	3.9	2.3
* **Plasma Protein Binding (%)**	7.6	

*Hernandez et al. AAC[23]

We further assessed the combination of epetraborole with ceftazidime *in vivo*. Epetraborole and ceftazidime were co-delivered at 30 mg/kg-200 mg/kg, 15 mg/kg-125 mg/kg, or 5 mg/kg-25 mg/kg combinations. These doses of oral epetraborole were chosen not to saturate the efficacy window in this model, thus allowing us to see at least additive efficacy from the combination of epetraborole and ceftazidime. The co-delivery of epetraborole orally at 30 and 15 mg/kg doses of BID and ceftazidime at 125 mg/kg or 200 mg/kg (SC), respectively, resulted in a ~ 5 Log_10_ CFU/mL reduction in bacterial burden in the lung compared to untreated control (**Fig 2E**). Epetraborole was also effective against disseminated disease in the spleen when delivered orally at 15 or 30 mg/kg as a single agent or combined with 125 or 200 mg/kg ceftazidime (SC) (**Fig 2F**).

**Fig 3:**
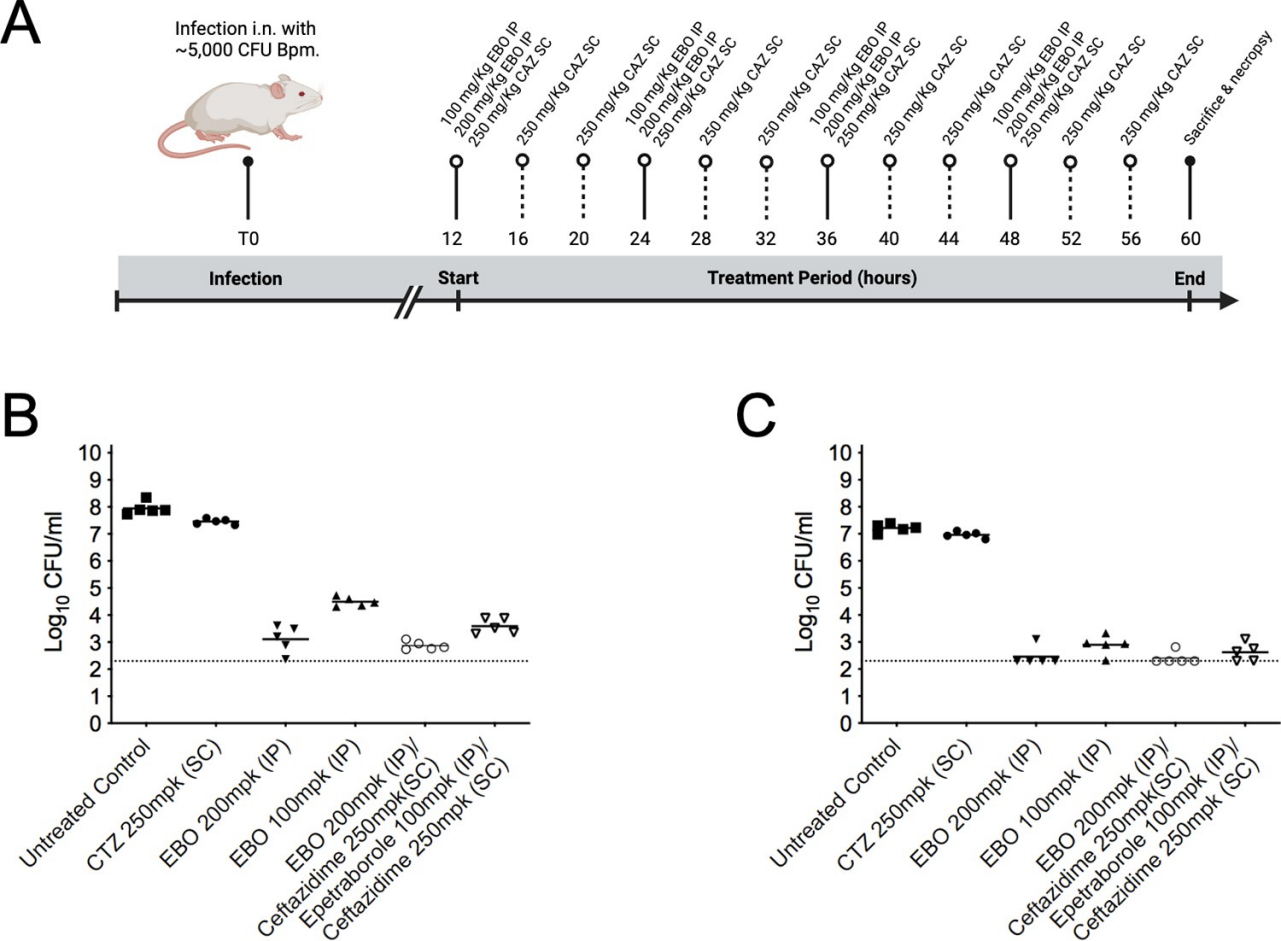
Delayed dosing with epetraborole against clinically derived *B*. *pseudomallei* strain. Dosing schedule of treatments in the delayed *B*. *pseudomallei* animal infection model (A). Bacterial burdens in the lung (B) and spleen (C) resulting from delayed treatment with CAZ and EBO delivered subcutaneously (SC), and intraperitoneally (IP), alone or in combination. Lower level of detection is indicated by dotted lines. Created with BioRender.

To further validate the potential improvement in clinical outcomes of epetraborole efficacy against the clinically derived strain MSHR435, which has elevated ceftazidime MIC values of 8 μg/mL, was investigated in a delayed dosing model where mice were dosed 12 hrs post-infection (**Fig 3A)**. Delayed dosing with epetraborole 100 mg/kg and 200 mg/kg IP BID significantly reduced the bacterial burden in the lung by ~ 3.4 and ~ 4.8 Log_10_ CFU/mL (**Fig 3B**)and in the spleen by ~ 3.8 and 4.8 Log_10_ CFU/mL, respectively (**Fig 3C**). Combining the ceftazidime dose regimen with 100 mg/kg epetraborole treatment enhanced the activity compared to 100 mg/kg epetraborole alone. Together, these studies demonstrate that epetraborole is efficacious against *B*. *pseudomallei* when delivered by different routes and offers an additive activity when combined with the SoC drug ceftazidime, even when treatment is initiated after infection.

## Discussion

Given the high unmet medical need for melioidosis, one of the goals in drug discovery is to identify candidates that can be used alone or in combination to treat this severe, rapidly progressing, potentially life-threatening disease. Once thought to only be endemic in Australia and Asia, recently, melioidosis has been isolated in the Americas at an increasing frequency, indicating that *B*. *pseudomallei* is more geographically widespread than previously thought [7], and, with potential environmental exposures, the number of patients with melioidosis is expected to rise. In addition, as of January 1^st,^ 2023, melioidosis has become a nationally notifiable urgent condition in the United States by the CDC [27]. The limited number of drugs effectively treating melioidosis and the long treatment times for a durable cure underscores the need for new therapies with unique modes of action that are not limited by drug efflux and transient antimicrobial resistance mechanisms [9,10]. A historically successful approach in drug discovery is repurposing or broadening the spectrum of a drug or new investigational drug candidate. Epetraborole is a boron-containing small molecule that inhibits LeuRS and has been shown in other nonclinical studies to have efficacy against medically important, difficult-to-treat bacterial pathogens [23]. Therefore, we assessed epetraborole against a diverse panel of laboratory and clinically derived *B*. *pseudomallei* strains and in animals of melioidosis to determine its potential as a clinical candidate that can be used alone or as a co-therapeutic to treat *B*. *pseudomallei* infections.

We have successfully used standardized strain panels to discover two new investigational drug candidates [28,29]. Similarly, we have used this screening approach to demonstrate that epetraborole has potency against various clinically derived *B*. *pseudomallei* strains with differing susceptibility to SoC drugs. An important metric of drug performance is the demonstrated comparable potency of epetraborole against *B*. *pseudomallei* strains with a spectrum of drug susceptibilities. Multiple encoded efflux systems have been well documented to impact the natural resistance rate of *B*. *pseudomallei* strains because even slight changes in more than one efflux activity would increase the required inhibitory concentration of the drug. Notably, the in vitro activity of epetraborole is minimally impacted by drug efflux that is well known to result in widespread drug resistance in *B*. *pseudomallei*. To assess the efficacy potential of epetraborole, the intracellular activity was evaluated using an *ex vivo* efficacy model routinely used in drug discovery efforts. Epetraborole was highly effective in significantly reducing the bacterial numbers in a dose-dependent manner by several log_10_ CFU. The activity in this *in vitro* assay indicates that epetraborole is very effective in killing intracellular bacteria in macrophages. The observed activity in an *ex vivo* model substantiates the ability of epetraborole to permeate the mammalian cell membrane and maintain activity in the host environment.

This study demonstrated that epetraborole possesses antibacterial activity against a panel of *B*. *pseudomallei* strains, including clinical strains with various susceptibilities to current SoC drugs, and has efficacy in an *ex vivo* model and a standard lethal mouse models of melioidosis. Epetraborole reduced the bacterial load in the lungs in a dose-dependent fashion. Notably, epetraborole demonstrated efficacy when administrated by different routes at equivalent doses. Consistent with the significant reduction in bacterial load in the lungs, the bacterial load in the spleen was typically at the level of detection or below. Given that 25% of patients on IV SoC therapy die within 1 month, with even a greater amount in 1 year, any improvement in SoC is desperately needed [30,31]. Therefore, epetraborole was assessed as a co-therapeutic with the SoC drug ceftazidime to determine its use as a new combination therapy regimen. The improved efficacy when epetraborole is co-administered with ceftazidime indicates that epetraborole has excellent potential as a therapeutic that can be used in combination with ceftazidime and adding epetraborole to treatment regimens could help improve outcomes when treating *B*. *pseudomallei* in the clinical setting. Even when epetraborole administration was delayed, it demonstrated efficacy against a clinically derived difficult-to-treat *B*. *pseudomallei* strain. These observations are particularly significant given the various intrinsic resistance and drug efflux mechanisms of *B*. *pseudomallei*. The potency of epetraborole against clinically derived strains and *in vivo* efficacy demonstrates the potential for epetraborole to improve clinical outcomes in combination with ceftazidime.
